# Exceptional CO_2_ Hydrogenation to Gasoline Enabled by GaZrO_x_/Ga‐ZSM‐5 Tandem Catalyst

**DOI:** 10.1002/advs.76074

**Published:** 2026-06-16

**Authors:** Wenhui Li, Yujing Sun, Bingyu Liu, Yaoyang Ni, Hong Yang, Xinwen Guo

**Affiliations:** ^1^ State Key Laboratory of Fine Chemicals Frontier Science Center For Smart Materials PSU‐DUT Joint Center For Energy Research School of Chemical Engineering Dalian University of Technology Dalian China; ^2^ School of Engineering The University of Western Australia Perth Western Australia Australia

**Keywords:** CO_2_ Hydrogenation, gallium Hydrides, GaZrO_x_, Ga‐ZSM‐5, tandem Catalyst

## Abstract

CO_2_ hydrogenation to gasoline offers a promising route for addressing energy challenges and enabling the effective utilization of carbon resources. However, the efficient conversion of CO_2_ to gasoline remains a significant challenge due to insufficient hydrogen activation capacity on existing metal oxide/zeolite tandem catalysts, as well as low selectivity of gasoline among the reaction products. In this work, we develop a tandem catalytic system comprising GaZrO_x_ and Ga‐substituted ZSM‐5 (Ga‐ZSM‐5) that achieves high CO_2_ conversion and high gasoline yield in CO_2_ hydrogenation. The optimized tandem catalyst, GZO‐700/Ga‐Z5(50), achieves a CO_2_ conversion of 17.2% with a C_5+_ selectivity of 83.2% at 320°C and 3 MPa, while maintaining low selectivities for CH_4_ (< 1%) and CO (11.7%). The catalyst shows no significant deactivation over 400 h of continuous operation. By tuning the calcination temperature, the GaZrO_x_ component is optimized to exhibit appropriate Ga‐H coverage and O‐vacancy concentration, which synergistically promotes H_2_ dissociation, H_2_ migration, and CO_2_ activation, leading to enhanced CO_2_ conversion and gasoline production. Furthermore, the incorporation of Ga into the ZSM‐5 framework in place of Al effectively reduces the acid strength and Brønsted/Lewis acid ratio while increasing the acid density, thereby significantly boosting the formation of C_5+_ hydrocarbons. The decrease in the acid strength of the catalyst also suppresses carbon deposition and, thus results in more than a twofold increase in the catalyst lifetime.

## Graphical abstract

A GaZrO_x_/Ga‐ZSM‐5 tandem catalyst achieves 17.2% CO_2_ conversion with 83.2% C_5+_ selectivity and >400 h stability relies on optimized O‐vacancies and Ga‐H coverage for CO_2_ hydrogenation.

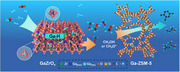

## 1 Introduction

CO_2_ hydrogenation to gasoline represents a promising pathway within Power‐to‐Liquid (PtL), aiming to transform carbon dioxide into high value‐added chemicals using green hydrogen [1, 2, 3]. It typically proceeds via two main routes: a direct pathway combining the reverse water‐gas shift (RWGS) reaction with Fischer–Tropsch synthesis (FTS) [4, 5], or an indirect methanol‐mediated pathway, where methanol is first synthesized from CO_2_ and subsequently converted into gasoline‐range hydrocarbons via methanol‐to‐gasoline (MTG) or related processes [6, 7]. Both of these routes require a tandem catalytic system to achieve the best gasoline selectivity [8, 9, 10]. Since the products generated through the methanol‐mediated pathway are not limited by the ASF distribution, the research effort attempts to start from the methanol‐mediated pathway to obtain higher gasoline selectivity. In‐based metal oxide was employed as a good candidate in tandem with ZSM‐5 for CO_2_ hydrogenation to gasoline [11]. Further more, the In‐based metal oxide can also be combined with gallium‐substituted self‐pillared pentasil nanosheets, achieving higher C_5+_ selectivity [12]. However, the CO_2_ conversion of In‐based metal oxide catalysts is not satisfactory due to the insufficient activation capacity of H_2_ and the constraints of thermodynamic equilibrium conversion of CO_2_ to methanol. Additionally, indium tends to migrate to the surface of the zeolite and cover the active sites, leading to catalyst deactivation. Therefore, there is an urgent need to develop a more efficient catalyst that can simultaneously enhance CO_2_ conversion and gasoline selectivity while preventing migration‐induced catalyst deactivation [12, 13, 14]. In contrast to the readily mobile In species, Ga^3+^ can be stably doped into the ZrO_2_ lattice to form a homogeneous solid solution in the GaZrO_x_‐involved tandem system [15]. It can be employed in tandem with SAPO‐34 and SSZ‐13 zeolites for the production of light olefins and propane, delivering a high target‐product selectivity of around 80% and good catalytic stability, with no Ga species migrating to the zeolite surface [16, 17]. In addition, GaZrO_x_ solid solutions have shown considerable potential for CO_2_ activation and hydrogenation [18, 19]. The formation of a homogeneous solid solution can enhance O‐vacancy concentration, a key factor in activating CO_2_ molecule [20]. Meanwhile, gallium oxide (Ga_2_O_3_) plays a multifaceted role: it not only contributes to CO_2_ chemisorption but also exhibits a remarkable ability to activate H_2_ [21]. This capability is attributed to the unique property of Ga species to form Ga─H bonds, which facilitate the H_2_ dissociation [22, 23]. Nevertheless, the migration of hydrides along lattice oxygen atoms increases the possibility of hydride elimination to hydroxyl, further exacerbating the hydride shortage [24]. To prevent the loss of H species during migration, a higher concentration of O‐vacancy is required. Achieving the effective activation of H_2_, the catalyst needs to simultaneously satisfy the H_2_ dissociation and migration on the surface of catalyst [25].

Moreover, on the tandem catalysts, alleviating the carbon deposition deactivation on ZSM‐5 is also an important and urgent problem to be solved. The carbon deposition on HZSM‐5 mainly occurs at the strong Brønsted acid sites [26, 27, 28]. The generation of carbon deposition will also correspondingly reduce the selectivity of target product C_5_‐C_11_ hydrocarbons. Therefore, to achieve higher efficiency in CO_2_ conversion to gasoline, attention should be paid to both the activation capacity of the metal catalyst for CO_2_ and H_2_ and the acidic properties of the H‐ZSM‐5 zeolite. In the H‐ZSM‐5 framework, Al^3+^ substitutes Si^4+^ to generate a negative charge, which is balanced by protons (H^+^), forming acidic sites Si‐O(H)‐Al. Replacing Al with other ions and modulating the T─O bonds (T = Si, Al, Ga) is one of the effective methods to change local electron density and alter the HZSM‐5 acidity [29, 30]. The proton affinity at the Ga^3+^ site is higher than that at the Al^3+^ site and the frequency of O─H stretching vibration shifts to higher wavenumbers [31, 32]. Replacing Al^3+^ with Ga^3+^ alters the electron distribution around the bridging hydroxyl group, stabilizing the O─H bond and making it less prone to dissociation to release a proton (H^+^) [33]. Kramer et al. calculated proton affinity energies for various bridged hydroxyl configurations (Si‐O(H)‐M) unequivocally and demonstrated that Ga‐ZSM‐5 exhibits a higher proton affinity than Al‐ZSM‐5 [34]. A greater proton affinity indicates enhanced O─H bond strength, resulting in weaker Brønsted acidity. Furthermore, the reduced Brønsted acidity can inhibit the secondary cracking of gasoline‐range hydrocarbons, thereby further enhancing gasoline selectivity. Therefore, substituting Al^3+^ with Ga^3+^ in ZSM‐5 is expected to reduce the acid strength of ZSM‐5, resulting in acidic properties more favorable for the CO_2_‐to‐gasoline reaction and prolong the catalysts lifetime.

In this work, we designed a GaZrO_x_ solid solution to modulate the Ga‐H coverage and O‐vacancy to enhance hydrogen and CO_2_ activation capacity, thereby obtaining the superior CO_2_ conversion. Through Ga substitution of Al atoms in ZSM‐5, the acidic properties of the catalyst are modified, leading to increased selectivity for gasoline products and retarded catalyst deactivation. Through a combination of catalytic testing and in situ spectroscopy, we seek to provide direct evidence for the Ga effects on CO_2_ and H_2_ activation and its synergy with ZSM‐5 for C_5+_ hydrocarbons generation, contributing to the rational design of next‐generation oxide catalysts for efficient CO_2_ to gasoline.

## 2 Results and Discussion

In this study, tandem catalysts GaZrO_x_/ZSM‐5 are designed, synthesised and eveluated for CO_2_ hydrogenation to gasoline. They are designated as GZO‐t/Z5(y) or GZO‐t/Ga‐Z5(y), where GZO stands for GaZrO_x_, Z5 stands for Al‐ZSM‐5, Ga‐Z5 stands for Ga‐ZSM‐5, t represents the calcination temperature of GaZrO_x_ and y represents the Si/Al ratio of ZSM‐5 or the Si/Ga ratio of Ga‐ZSM‐5.

### 2.1 Achieving Exceptional CO_2_ Hydrogenation to Gasoline Over GaZrO_x_/ZSM‐5 Tandem Catalysts

**FIGURE 1:**
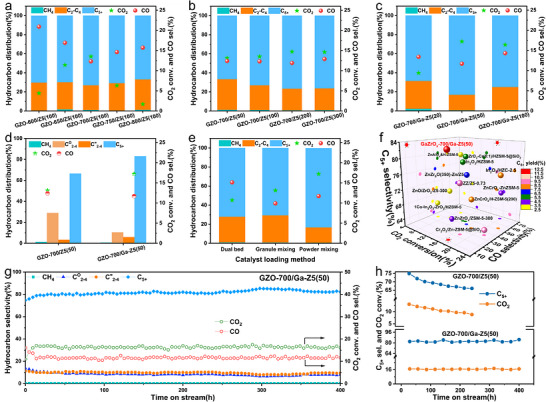
The catalytic performance of (a) GaZrO_x_ calcined at different temperatures combined with ZSM‐5. (b) GZO‐700 combined with ZSM‐5 of different Si/Al ratios. (c) GZO‐700 combined with Ga‐ZSM‐5 of different Si/Ga ratios. (d) Catalytic performance comparison of GZO‐700/Ga‐Z5(50) and GZO‐700/Z5(50). (e) The catalytic performance of GZO‐700/Ga‐Z5(50) with different filling methods. (f) Comparison of the catalytic performance of GZO‐700/Ga‐Z5(50) catalyst with the previously reported catalysts for CO_2_ hydrogenation to C_5+_ through the methanol‐mediated pathway. (g,h) Stability tests for GZO‐700/Z5(50) and GZO‐700/Ga‐Z5(50).

Figure 1 presents the CO_2_ hydrogenation to gasoline reaction results under conditions of 320°C, 3 MPa, and 3600 mL·g^−1^·h^−1^ of gaseous hourly space velocity over tandem catalysts GZO‐t/Z5(100) (t = 600–800°C). As seen in Figure 1a and Table S1, at low calcination temperature of 600°C, CO_2_ conversion and C_5+_ selectivity are 4.5% and 70.3%, respectively. As the calcination temperature increases, the CO_2_ conversion and C_5+_ selectivity gradually rise and reach the maximum values 13.5% and 73.0%, respectively over GZO‐700/Z5(100), after which they begin to decline. When the calcination temperature reaches 800°C, the CO_2_ conversion decreases to 1.7%, and the selectivity of gasoline products also decreases to 66.5%. It is worth noting that the selectivity of CO decreases initially and then increases with the increase in calcination temperature, which reaches its minimum 12.5% on GZO‐700/Z5(100). The CO_2_ conversion and C_5+_ selectivity are also obtained on catalysts where GZO‐700 is coupled with ZSM‐5 of different Si/Al ratios from 50 to 300 (Figure 1b and Table S2). The highest CO_2_ conversion and C_5+_ selectivity of 14.7% and 76.5% are achieved on GZO‐700/Z5(200). Detailed products distribution of C_5+_ products on GZO‐700/Z5(200) for CO_2_ hydrogenation is shown in Table S3. The C_5+_ products are mainly composed of aromatics, among which C_5,_ C_6_ and C_7_ hydrocarbons are mainly aliphatics, and C_8_‐C_11_ hydrocarbons are mainly methylbenzene. Among the aromatic hydrocarbons, the selectivity of 1,2,4,5‐Tetramethylbenzene is the highest.

Furthermore, Ga‐ZSM‐5 (Si/Ga = 20, 50, 100) catalysts are also evaluated. As seen in Figure 1c and Table S4, at Si/Ga = 50, the CO_2_ conversion and C_5+_ selectivity reach the maximum of 17.2% and 83.2%, respectively, while maintaining low selectivities for CH_4_ (< 1%) and CO (11.7%). Compared to GZO‐700/Z5(50), GZO‐700/Ga‐Z5(50) shows obvious improvements on both CO_2_ conversion and C_5+_ selectivity (Figure 1d). At the same time, the generation of C_2_‐C_4_ alkanes is reduced on GZO‐700/Ga‐Z5(50). This study has also investigated three configurations for integrating GZO‐700 with Ga‐Z5(50) (Figure 1e and Table S5). It has been found that the CO_2_ conversion is the lowest but the CO selectivity is the highest in a sequential GZO‐700/Ga‐Z5(50) double‐bed configuration. In a mixing‐particle configuration, CO_2_ conversion increases while the CO production decreases compared with the double‐bed configuration. The CO_2_ conversion and C_5+_ selectivity achieve the highest in the mixing‐powder configuration. It seems that intimate oxide/zeolite powder mixing promotes the migration of intermediates and generation of C_5+_ hydrocarbons. Compared with the reported catalysts for methanol‐mediated CO_2_ hydrogenation to C_5+_ hydrocarbons, the GZO‐700/Ga‐Z5(50) catalyst shows the highest C_5+_ selectivity and yield due to the high CO_2_ conversion and low CO selectivity (Figure 1f and Table S6).

Finally, catalytic stability tests are conducted on GZO‐700/Ga‐Z5(50) and GZO‐700/Z5(50) both prepared in powder‐mixing configuration (Figure 1g,h). As seen, on GZO‐700/Ga‐Z5(50) catalyst the CO_2_ conversion remains at ∼17% and C_5+_ selectivity at ∼82% upto 400 h, with no significant deactivation. In contrast, on GZO‐700/Z5(50), after 150 h, the CO_2_ conversion and C_5+_ selectivity significantly decrease and drop to 8.8% and 66%, respectively at 240 h. The thermogravimetric (TG) data of the two spent catalysts were show in Figure S1. Seen from the TG curve, the weight loss above 300°C is regarded as the elimination of carbon deposits. By comparing Figure S1a,b, it can be seen that after 400 h on stream, the weight loss of spent GZO‐700/Ga‐Z5(50) is 1.98 wt% with 0.049 mg_coke_·g_cat_^−1^·h^−1^ carbon deposition rate, while after 240 h on stream, the weight loss of spent GZO‐700/Z5(50) is 1.67wt% with 0.069 mg_coke_·g_cat_^−1^·h^−1^ carbon deposition rate. The carbon deposition rate of Ga‐Z5(50) is significantly reduced by approximately 29% compared with that of Z5(50).

### 2.2 Revealing the Fine Structure and Generating O‐Vacancies on GaZrO_x_ for CO_2_ Activition

**FIGURE 2:**
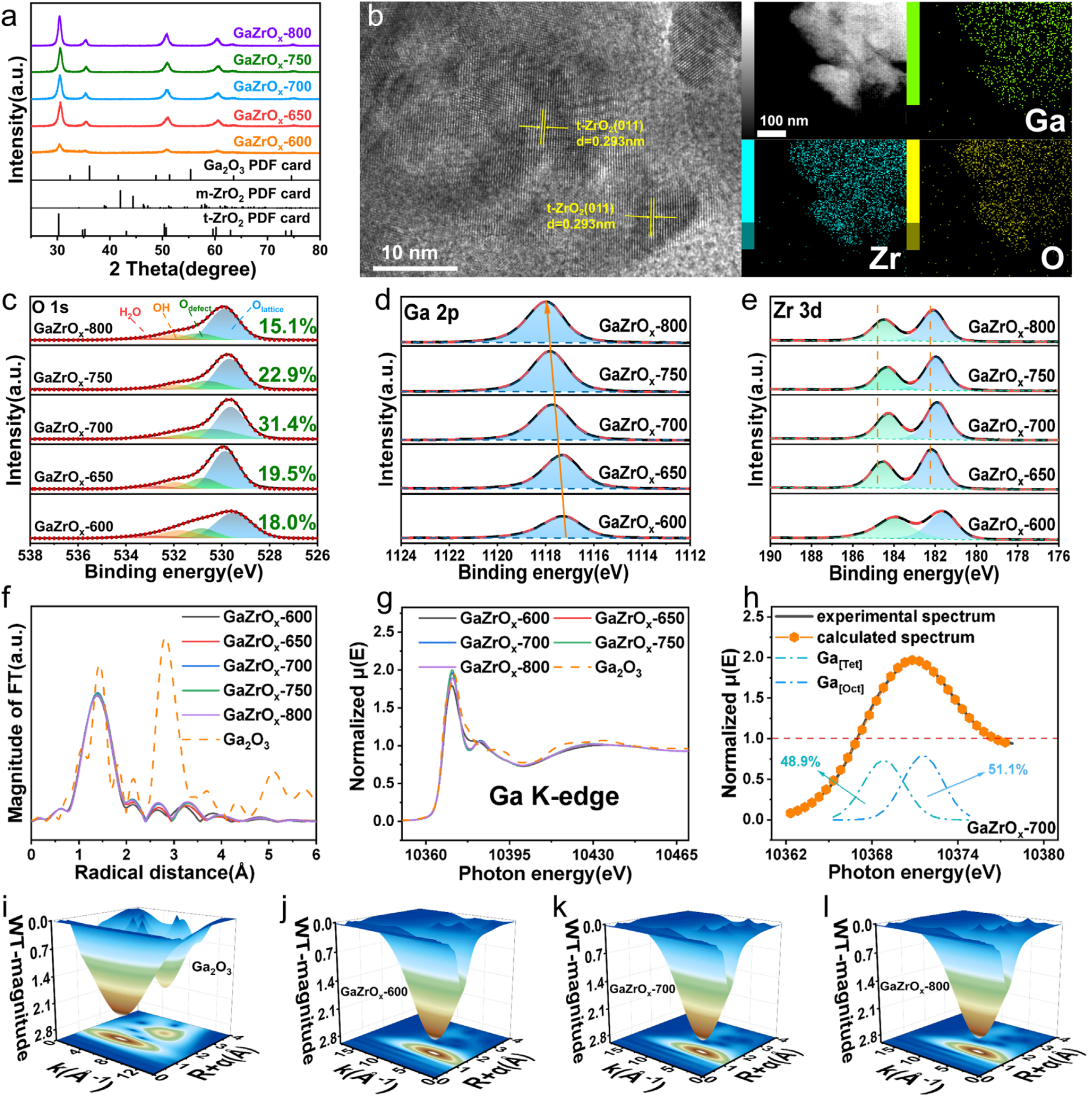
Structural characterization of different GaZrO_x_ solid solutions. (a) XRD patterns of GaZrO_x_ samples. (b) STEM images and mapings of GZO‐700. (c) O 1s XPS spectra. (d) Ga 2p XPS spectra. (e) Zr 3d XPS spectra. (f) Fourier transform of the Ga K‐edge EXAFS spectra for the GaZrO_x_ catalysts and the reference Ga_2_O_3_ under ambient conditions. (g) Ga K‐edge XANES spectra. (h) The Ga K‐edge XANES spectra of the GZO‐700 sample and its simulated spectra, which consists of two types of Ga^3+^ (tetrahedral tetra‐coordinated Ga_[Tet]_ and octahedral hexa‐coordinated Ga_[Oct]_). (i–l) k^3^‐weighted wavelet transforms of the Ga K‐edge EXAFS spectra of different samples.

Figure 2 summersies the structure characterization results of GaZrO_x_ (GZO‐t) samples. First, x‐ray diffraction (XRD) patterns (Figure 2a) of GaZrO_x_ samples synthesized at different calcination temperatures (600–800°C) display only diffraction peaks at 30.3°, 35.5°, 50.8°, 60.5°, and 63.1°, all of which are indexed to the tetragonal ZrO_2_ phase (t‐ZrO_2_, PDF#50‐1089). No diffraction peaks corresponding to monoclinic ZrO_2_ (m‐ZrO_2_, PDF#37‐1484) or Ga_2_O_3_ are detected. Examining the diffraction pattern closely, the diffraction peaks of GZO‐700 (Figure S2) all shift to higher 2θ angles relative to those of standard t‐ZrO_2_. These results seem to indicate that the prepared GaZrO_x_ samples are of a single phase with the t‐ZrO_2_ crystal lattice of a degree of lattice distortion caused by the substitution of Ga^3+^ of Zr^4+^ [35, 36].

Scanning transmission electron microscopy (STEM) imaging of GZO‐700 shows the lattice spacing close to d_(011)_ of standard t‐ZrO_2_ (Figure 2b). EDS elemental mapping also shows uniform distributions of Ga, Zr, and O elements throughout GZO‐700 particles (Figure 2b), which agrees with the single phase conclusion for GaZrO_x_. Ar adsorption–desorption isotherms of the GaZrO_x_ samples (Figure S3) display a type‐IV behavior with H2‐type hysteresis loop, confirming the presence of a mesoporous structure. The specific surface area initially increases and then decreases with the rising calcination temperature (Table S7). When the temperature increases from 600°C to 650°C, the surface area increases. Combined with thermogravimetric analysis (TGA) results (Figure S4), the lower surface area of the sample calcined at 600°C is attributed to residual carbonaceous species formed from the incomplete decomposition of glucose at lower temperatures. As the temperature is raised to 650°C, the carbon species are gradually removed, leading to an increase in surface area.

The surface electronic states of GaZrO_x_ are examined by x‐ray photoelectron spectroscopy (XPS) (Figure 2c,d). The O 1s spectra (Figure 2c) reveal the presence of four distinct O‐species on the catalyst surface: lattice oxygen (O_lattice_) with binding energies between 529.5 and 529.9 eV, surface‐adsorbed oxygen at defect sites (O_defect_) between 530.1 and 530.9 eV, surface hydroxyl groups (O_OH_) between 531.6 and 531.9 eV, and oxygen from adsorbed water molecules (O_H2O_) between 532.8 and 533.0 eV [16, 37]. The O_defect_ fractions in GaZrO_x_ are listed in Figure 2c and the fractions of all type of oxygen species are provided in Table S8. Among the samples, GZO‐700 exhibits the highest concentration of O‐vacancies of 30.5%. When the calcination temperature is too low, residual precursors hinder the formation of O‐vacancies. Conversely, excessively high calcination temperatures accelerate the diffusion rate of oxygen ions within the system, making it easier for external oxygen atoms to embed into the crystal lattice and occupy defect sites, thereby reducing the concentration of O‐vacancies. Thus, the optimal calcination temperature promotes the formation of surface O‐vacancies.

CO_2_‐temperature‐programmed desorption (CO_2_‐TPD) profiles (Figure S5) show a desorption peak in the range of 50–150°C, indicating a moderate‐strength interaction between CO_2_ and these vacancy sites. The highest concentration of O‐vacancies in GZO‐700 is conducive to the adsorption and activation of CO_2_, thus GZO‐700 exhibits the highest CO_2_ conversion (Figure 1a). In Ga 2p_3/2_ spectra, the peak at 1117.4 eV assigned to Ga^3+^ species (Figure 2d) [38]. The Zr 3d spectra (Figure 2e) shows two characteristic peaks at 181.9 and 184.3 eV, attributed to Zr 3d_5/2_ and Zr 3d_3/2_ of Zr^4+^ species, respectively [39]. Notably, upon the incorporation of Ga into ZrO_2_, the Ga 2p_3/2_ peaks shift to higher binding energies, while the Zr 3d peaks shift to lower binding energies as the calcination temperature increases from 650°C to 800°C. This observation suggests the electron density around Ga^3+^ decreases, while that around Zr^4+^ increases [15, 40].

H_2_‐TPR was employed to evaluate the reducibility of the GaZrO_x_ catalysts (Figure S6). The results demonstrate that GaZrO_x_ is not readily reduced at temperatures ≤ 600°C. When the temperature is above 600°C, the reduction peak is attributed to the more O‐vacancies formation. Combined with XPS data, this confirms that Ga^3+^ maintains a stable oxidation state under reaction conditions (320°C). Notably, the reduction peak for GZO‐600 is significantly more intense than those for catalysts calcined at higher temperatures, which is attributed to the concurrent decomposition of residual carbon species during the high‐temperature reduction.

X‐ray absorption fine structure (XAFS) spectroscopy is used to probe the local coordination environments of Ga and Zr in GaZrO_x_. The Zr K‐edge XANES spectra (Figure S7a) of GaZrO_x_ samples show nearly identical absorption edges to that of pure ZrO_2_, confirming the Zr^4+^ oxidation state. The Fourier‐transformed EXAFS (FT‐EXAFS) spectra of GaZrO_x_ and a ZrO_2_ reference (Figure S7b) exhibit peaks in the first and second coordination shells, corresponding to Zr‐O and Zr‐Zr backscattering paths, respectively. It is worth noting that the bond length of Zr‐Zr has slightly increased in GaZrO_x_ catalysts than ZrO_2_, indicating that Ga has entered the ZrO_2_ lattice and resulted the lattice distortion of ZrO_2_. Further quantitative fitting of EXAFS (Table S9 and Figures S8–S10) revealed that the coordination number of Zr‐O was close to 7 and did not change significantly with the increase in the calcination temperature of the catalyst. This was significantly lower than the Zr‐O coordination number of t‐ZrO_2_, which was 8 [41, 42]. In order to maintain charge balance under the replacement of Ga^3+^ to Zr^4+^, O‐vacancies are generated and concentrated near Zr^4+^ [43]. As O‐vacancies are structural defects, they directly disrupt the complete coordination of Zr‐O, which is consistant with the XPS results (Figure 2c).

Both GaZrO_x_ catalysts and the Ga_2_O_3_ reference show a distinct peak in the first coordination shell (Ga‐O) in the FT‐EXAFS spectra (Figure 2f). A second‐shell peak corresponding to Ga‐Ga scattering is prominent in Ga_2_O_3_ but is absent or very weak in all GaZrO_x_ samples. The Ga K‐edge XANES spectra (Figure 2g) display absorption edges similar to that of pure Ga_2_O_3_, confirming the +3 oxidation state of Ga. A more pronounced white‐line intensity (1s→4p transition) is observed for samples calcined at higher temperatures (700°C and 750°C), reflecting a greater degree of unoccupied states for Ga in GaZrO_x_ [38].

The quantitative fitting results of EXAFS (Table S10 and Figures S11 and S12) show that, with the increase in calcination temperature, the average coordination number of Ga^3+^ first increases from 4.5 to 5.0 and then remains stable. The coordination environment of Ga^3+^ in the GZO‐700 sample was characterized by linear fitting of X‐ray absorption near‐edge structure (XANES) (Figure 2h). The absorption peaks near 10369 and 10372 eV correspond to tetrahedral tetra‐coordinated Ga^3+^ (Ga_[Tet]_) and octahedral hexa‐coordinated Ga^3+^ (Ga_[Oct]_) with the 1:1 ratio [23, 44]. It was reported [23] that tetra‐coordinated Ga^3+^ (Ga_[Tet]_) promotes the heterolytic dissociation of hydrogen, while hexa‐coordinated Ga^3+^ (Ga_[Oct]_) promotes the homolytic dissociation of hydrogen. The homolytic dissociation of H_2_ occurs after the heterolytic dissociation and will significantly enhance the ability of GaZrO_x_ for H_2_ dissociation.

A distinct peak in the second coordination shell of the Ga_2_O_3_ standard sample corresponds to backscattering of Ga‐Ga, while the feature in the same region of GaZrO_x_ is not obvious. Therefore, a k^3^‐weighted wavelet transform was performed on the Ga K‐edge EXAFS spectra of the samples (Figures 2i–l and Figure S10). The k^3^‐weighted EXAFS spectra show the main features in R‐space and k‐space, further indicating that there is no Ga_2_O_3_ in GaZrO_x_. This phenomenon is consistent with the XRD results, further demonstrating that Ga atoms are highly dispersed in the GaZrO_x_ solid solution and O‐vacancies are produced by lattice distortion.

### 2.3 Modulating Ga‐H Intensity for H_2_ Activation on GaZrO_x_

**FIGURE 3:**
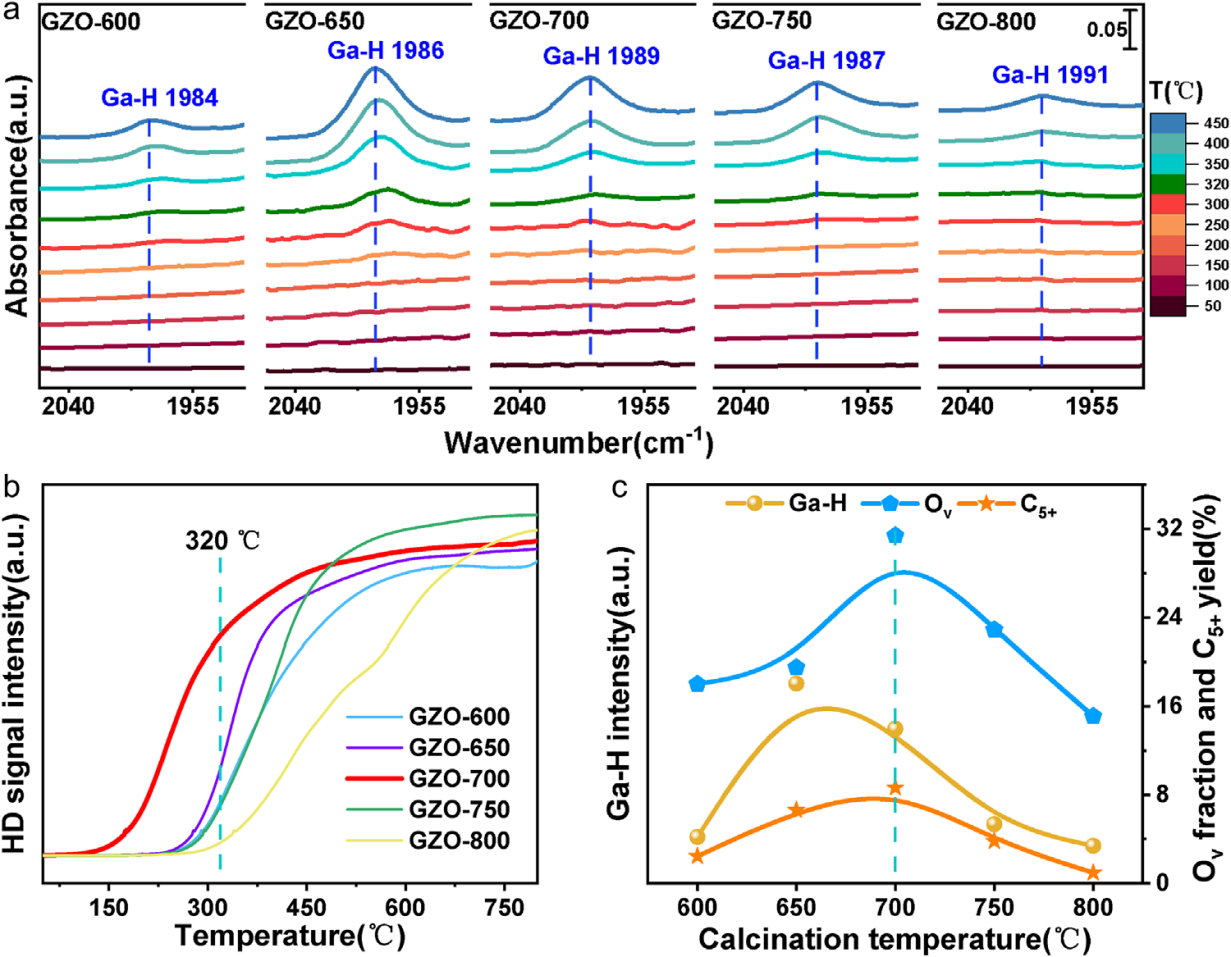
Investigation of Hydrogen Activation and Dissociation on GaZrO_x_. In situ DRIFTS spectra of (a) GaZrO_x_ under pure hydrogen atmosphere at 3 MPa recorded from 50°C to 450°C. (b) H_2_‐D_2_ exchange profiles of GaZrO_x_ solid solutions calcined at different temperatures. (c) Correlations between surface O‐vacancy, Ga‐H intensity, and C_5+_ yield on tandem catalysts.

Figure 3 presents the Diffuse Reflectance Infrared Fourier Transform Spectroscopy (DRIFTS) analysis and H_2_‐D_2_ exchange results of GaZrO_x_. Hydrogen activation behaviour on GaZrO_x_ is probed by in situ DRIFTS under pure H_2_ atmosphere. As seen, increasing the testing temperature leads to the development of a distinct peak at ∼1985 cm^−1^ for all samples. This peak is associated with Ga‐H stretching vibration [45]. The greater intensity of the Ga‐H peak indicates a higher coverage of Ga‐H species on the catalyst surface. Notably, the Ga‐H coverage on GZO‐650 is higher than that on GZO‐700 (Figure 3a).

The Ga‐H species serve as an indicator of the capability to facilitate H_2_ dissociation, while the ability to form active H species during the reaction is further investigated by H_2_‐D_2_ exchange experiments (Figure 3b). As seen, the HD signal intensity for all GaZrO_x_ samples increases with the rising temperature before leveling off, indicating a thermally enhanced hydrogen activation process where the generation of active hydrogen species progressively increases until saturation. Notably, GaZrO_x_ exhibit significant differences in their hydrogen activation behaviors. On GZO‐700, HD starts to form at ∼150°C, whereas other GZO‐t catalysts begin to produce HD only at ∼250°C. At 320°C reaction temperature, GZO‐700 shows the strongest HD signal, confirming its superior hydrogen activation and dissociation capacity.

A trend graph is constructed to show the relationship between the Ga‐H peak intensity and the catalytic performance of GZO‐t/Z5(100) tandam catalysts (Figure 3c). As seen, GZO‐700/Z5(100) delivers the highest CO_2_ conversion and C_5+_ yield with appropriate Ga‐H coverage and the highest O‐vacancy concentration. The GZO‐650 sample has the largest Ga‐H coverage, but its H activation ability is inferior to that of the GZO‐700 sample, which is associated with the highest O‐vacancy concentration on GZO‐700. It was reported that the hydrogen species migrating along lattice oxygen tend to be captured and converted into inactive hydroxyl groups (─OH), resulting in hydride elimination and the loss of hydrogen reactivity. In contrast, oxygen vacancies provide exclusive low‐barrier migration pathways for active hydrogen. Hydride ions migrate rapidly between adjacent oxygen vacancies while retaining their reactive hydridic state, without being converted into hydroxyl species [24, 46]. The higher oxygen vacancy concentration is highly beneficial for stabilizing active hydrogen species, and promoting efficient hydrogen migration for CO_2_ activation. Therefore, for the activation of H_2_, the first step is to dissociate H_2_ with a high Ga‐H coverage, and the second is to enable the generated H species to migrate on the catalyst surface. Only the H species that meet both conditions simultaneously can be regarded as the activated H species that contribute to CO_2_ hydrogenation. In situ DRIFTS under the reaction conditions are also conducted (Figure S13). The absorption peaks at 1580–1590^−^ and 1350–1360 cm^−1^ correspond to the HCOO^*^ species formed by asymmetric and symmetric stretching of v(O─C─O) [47]. On GZO‐700, the highest proportion of HCOO^*^ is observed, which is the key intermediate for CO_2_ to gasoline and inhibits the generation of CO [15, 48, 49, 50]. Effective H_2_ activation for CO_2_ hydrogenation requires not only high Ga‐H coverage for H_2_ dissociation but also sufficient O‐vacancies to facilitate hydride migration, with GZO‐700/Z5 achieving an optimal balance between O‐vacancies and Ga‐H coverage that maximizes the CO_2_ conversion for gasoline synthesis.

### 2.4 Regulating Acid Properties by Ga‐ZSM‐5 for Gasoline Selectivity

**FIGURE 4:**
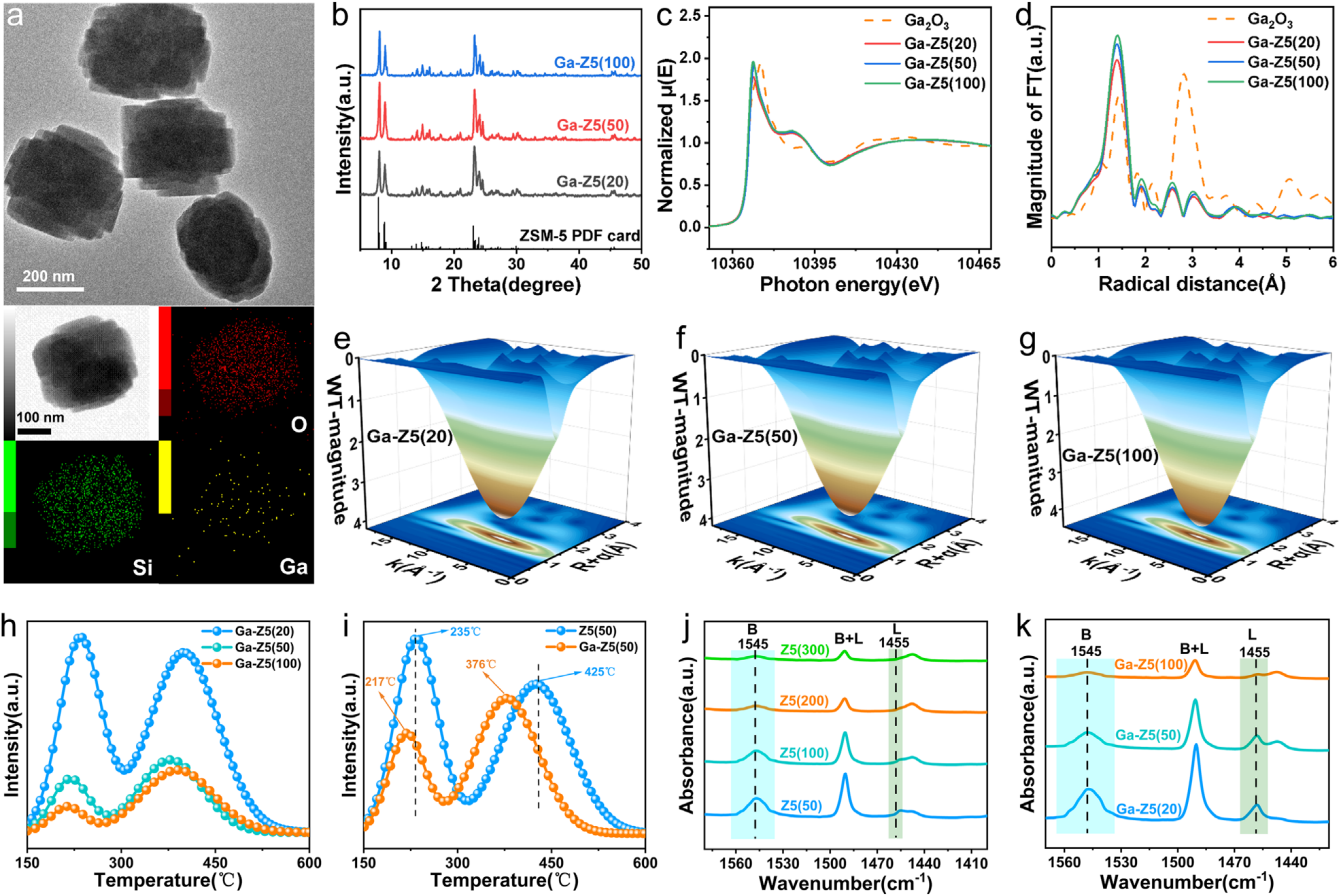
Structural and acid property investigation of ZSM‐5 and Ga‐ZSM‐5. (a) STEM images and EDS element maps of Ga‐Z5(50) catalyst. (b) XRD patterns of Ga‐ZSM‐5. (c) Ga K‐edge XANES spectra and (d) Ga K‐edge EXAFS spectra of different Si/Ga ratios on Ga‐ZSM‐5. (e–g) k^3^‐weighted wavelet transforms of the Ga K‐edge EXAFS spectra of different Ga‐Z5 samples. (h) NH_3_‐TPD profiles of Ga‐ZSM‐5 with different Si/Ga ratios. (i) Comparison of acid strength between Ga‐Z5(50) and Z5(50). (j) Py‐IR profiles of ZSM‐5 samples at 150°C. (k) Py‐IR profiles of Ga‐ZSM‐5 samples at 150°C.

The CO_2_ conversion mainly depends on the activation of H_2_ and CO_2_ by GaZrO_x_, while the regulation of gasoline selectivity mainly relies on the acidic propertity on ZSM‐5. Therefore, we designed Ga‐ZSM‐5 with Ga replacing Al to tailor the acid properties. Figure 4 presents the structural and acid property of the synthesized ZSM‐5 and Ga‐ZSM‐5 samples of different Si/Al or Si/Ga ratios (Table S11). First, substituting Al of the regular ZSM‐5 with Ga to form Ga‐ZSM‐5 does not impact on the MFI topological structure, crystal morphology, and overall texture properties of the resulting Ga‐ZSM‐5 with the Si/Al and Si/Ga ratios studied. SEM analysis of the ZSM‐5 (Figure S14 a–d) and Ga‐ZSM‐5 (Figure S15 a–c) samples show that they all consist of regular elliptical crystals. XRD analysis of the samples shows that they all exhibit characteristic diffraction peaks corresponding to MFI topological structure (JCPDS No. 42‐0024) at 2θ = 7.79°, 8.69°, 23.08°, 23.48°, 23.78° and 24.28° (Figure S16 and Figure 4b). No other diffractions peaks are observed in these samples. Furthermore, nitrogen physical adsorption–desorption measurements of the samples show that they all display type I isotherms (Figures S17 and S18) typical of a microporous material. BET specific surface areas of the samples vary little with the Si/Al or Si/Ga ratio of the sample and remain at ∼ 440 m^2^/g (Tables S12 and S13). The pore structures also remain similar among these samples. In Figure S14, the Ga‐Z5(20) sample is composed of smaller particles, which leads to the larger external surface area compared with Ga‐Z5(50)(Table S13). Seen from Figure 1c, more acid sites on the external surface of ZSM‐5 leads to the cracking of hydrocarbons, and also significantly reduces the selectivity of the C_5_‐C_11_ products [51, 52]. Therefore, the low C_5+_ selectivity on the GZO‐700/Ga‐Z5(20) catalyst is related to the greater number of acidic sites on the external surface caused by the smaller particle size.

X‐ray absorption fine structure (XAFS) was used to characterize the valence state and coordination of Ga in the Ga‐ZSM‐5 sample. The x‐ray absorption near‐edge structure (XANES) spectra (Figure 4c) indicate that the absorption edge energy of Ga‐Z5 of different Si/Ga ratios is very similar. All samples show +3 valence of Ga because the absorption edge energy of all samples is approximately 10372 eV, which is similar to the value of the reported standard Ga^3+^ species [53, 54]. The Fourier transform of the k^3^‐weighted extended x‐ray absorption fine structure (EXAFS) spectra of Ga‐Z5 (Figure 4d) shows a Ga─O bond near 1.5 Å, and no Ga─Ga bond is observed. The k^3^‐weighted wavelet transformation of the Ga K‐edge EXAFS spectra of different Ga‐ZSM‐5 samples further confirms the absence of disordered clusters of Ga_2_O_3_ at this location (Figure 4e–g) [29]. To extract detailed local structure information from the Ga‐ZSM‐5 samples, quantitative analysis of their EXAFS spectra was performed, and the fitting results (Table S14, Figures S19 and S20) indicate that the Ga─O coordination number is around 4, with all bond lengths being 1.81 Å.

Ammonia Temperature‐Programmed Desorption (NH_3_‐TPD) profiles of the ZSM‐5 samples (Figure S21) display two NH_3_ desorption peaks at ∼230°C and ∼420°C, corresponding to the weak acid center and strong acid center, respectively. As Si/Al ratio increases, both peak areas under the curve decrease, indicating a reduction in both the weak and strong acid quantities in the sample. The NH_3_‐TPD profiles of the Ga‐ZSM‐5 samples (Figure 4h) also display two NH_3_ desorption peaks at ∼210°C (weak acid center) and ∼370°C (strong acid center). The acid quantity in Ga‐ZSM‐5 also decreases with the increase of the Si/Ga ratio of the sample. Plotting the NH_3_‐TPD profiles of Z5(50) and Ga‐Z5(50) together (Figure 4i), it is notable that first, the total acid quantity of Z5(50) is higher than that of Ga‐Z5(50), and second, both weak and strong acid centers are of lower acid strength compared to those in Z5(50) indicated by the lower desorption temperatures.

Brønsted/Lewis acid sites (BAS/LAS) of the samples are analysed by Pyridine‐infrared (Py‐IR) taken under vacuum at 150°C, where the desorption of Py molecules occurs at Brønsted acid sites (BAS) at ∼1545 cm^−1^, at Lewis acid sites (LAS) at ∼1455 cm^−1,^ or at combined Brønsted and Lewis sites at ∼1490 cm^−1^ [55, 56, 57]. From Py‐IR profiles of ZSM‐5 (Figure 4j) and Ga‐ZSM‐5 (Figure 4k), the acid properties of ZSM‐5 and Ga‐ZSM‐5 are determined (Table 1). As seen, with the increase of Si/Al or Si/Ga ratios, the acid strength and density of both Brønsted acid and Lewis acid decrease, but the B/L ratio does not change significantly. The B/L ratio of ZSM‐5 is generally larger than that of Ga‐ZSM‐5.

**TABLE 1:** The acid properties of ZSM‐5 and Ga‐ZSM‐5.

Samples	Acidity (µmol·g^−1^)			
	C_Brønsted_	C_Lewis_	Total	B/L
Z5(50)	135.5	15.0	150.3	9.0
Z5(100)	97.1	11.0	108.1	8.8
Z5(200)	48.2	5.8	54.0	8.3
Z5(300)	28.2	3.0	31.2	9.4
Ga‐Z5(20)	217.8	80.9	298.7	2.7
Ga‐Z5(50)	73.6	23.7	97.3	3.1
Ga‐Z5(100)	63.1	16.6	79.7	3.8

By tuning the acid properties through zeolite construction directed by gallium, an optimal balance of acid content, strength, and Brønsted‐to‐Lewis ratio can be achieved to maximize gasoline‐range hydrocarbon selectivity. The electronegativity of the Ga^3+^ site is higher than that of the Al^3+^ site, stabilizing the O─H bond and making it less prone to dissociation to release a proton (H^+^). Substituting Al^3+^ with Ga^3+^ in ZSM‐5 results in weaker Brønsted acid strength, which is more favorable for the CO_2_‐to‐gasoline and prolong the catalysts lifetime. The deactivation of GZO‐700/Z5(50) is mainly caused by carbon deposition on the ZSM‐5 zeolite. Methanol intermediates undergo oligomerization, cyclization, and hydrogen transfer reactions on the strong Brønsted acid sites, which block the pore channels and cover the active sites, resulting in catalyst deactivation. In contrast, the weakened Brønsted acidity of Ga‐Z5(50) can significantly suppress carbon deposition (Figure S1). In addition, seen from Figure 1c, the excessively high Brønsted acid density of Ga‐Z5(20) led to the cracking of long‐chain hydrocarbons, and the insufficient of Brønsted acid in Ga‐Z5(100) is unfavorable for the polymerization of low‐carbon hydrocarbons, causing a large amount of low‐carbon alkenes in the products. Compared with Z5(50), Ga‐Z5(50) has reduced acid strength while maintaining an appropriate acid content and B/L ratio, thereby the GZO‐700/Ga‐Z5(50) has the highest C_5+_ selectivity. This not only meets the kinetic requirements for C‐C coupling but also avoids excessive cracking of high‐carbon hydrocarbons. Therefore, by introducing Ga species to direct the zeolite construction, it efficiently promotes the generation of gasoline products.

## 3 Conclusion

This study successfully engineered a novel tandem catalyst for direct CO_2_ hydrogenation to gasoline with exceptional and stable performance. Under optimized reaction conditions (GHSV = 3600 mL·g^−1^·h^−1^, T = 320°C), the tandem catalyst GZO‐700/Ga‐Z5 achieved the highest activity and selectivity. Notably, it maintained stable operation for over 400 h on stream, delivering a steady CO_2_ conversion of 17.2% and gasoline‐range hydrocarbon selectivity of 83.2%.

Systematic variation of the calcination temperature enabled precise modulation of O‐vacancy concentration in the GaZrO_x_. In situ DRIFTS characterization revealed that GaZrO_x_ calcined at 700°C possesses moderate Ga‐H coverage for hydrogen dissociation. Consistently, in H_2_‐D_2_ exchange experiments, GaZrO_x_‐700 exhibited the lowest onset temperature for HD formation, confirming its superior H_2_ activation capability among the series. Effective H_2_ activation for CO_2_ hydrogenation requires not only high Ga‐H coverage for H_2_ dissociation but also sufficient O‐vacancies to facilitate hydride migration, achieving the highest CO_2_ conversion on GZO‐700/Z5. Gallium‐directed zeolite construction enables precise tuning of acid properties, yielding an optimal combination of acid content, strength, and Brønsted‐to‐Lewis ratio that maximizes the selectivity for gasoline‐range hydrocarbons. The composites of GaZrO_x_ and Ga‐ZSM‐5 were obtained and showed excellent catalytic performance for the hydrogenation of CO_2_ to gasoline. The balanced weak/strong and Brønsted/Lewis acidity imparted by Ga‐ZSM‐5 synergistically enhanced gasoline selectivity and prolonged catalyst stability. This work provides a compelling blueprint for the rational design of next‐generation tandem catalysts, transforming CO_2_ from a greenhouse gas into a valuable resource for clean energy production.

## Conflicts of Interest

The authors declare no conflicts of interest.

## Supporting information




**Supporting File**: advs76074‐sup‐0001‐SuppMat.docx.

## Data Availability

The data that supports the findings of this study are available in the Supplementary Material of this article.
