# Supplementary material for: Exceptional CO2 Hydrogenation to Gasoline Enabled by GaZrOx/Ga‐ZSM‐5 Tandem Catalyst
Source: Adv Sci (Weinh). 2026 Jun 16;13(50):e76074. doi: 10.1002/advs.76074 (PMC13336408; doi:10.1002/advs.76074)
Supplement: Supplementary file 1 [file ADVS-13-e76074-s001.docx]

**Supporting Information**

**Exceptional CO_2_ Hydrogenation to Gasoline Enabled by GaZrO_x_/Ga-ZSM-5 Tandem Catalyst**

Wenhui Li,^[a]^ Yujing Sun,^[a]^ Bingyu Liu,^[a]^ Yaoyang Ni,^[a]^ Hong Yang,^[a]^ Xinwen Guo*^[a]^

[a] State Key Laboratory of Fine Chemicals, Frontier Science Center for Smart Materials, PSU‐DUT Joint Center for Energy Research, School of Chemical Engineering, Dalian University of Technology, Dalian 116024, Liaoning, China, E-mail:guoxw@dlut.edu.cn
[b] School of Engineering, The University of Western Australia, Perth, WA 6009, Australia

**Table of Contents**

[1. Experimental section	1](#_Toc8358)

[2. Supporting Figures	5](#_Toc26073)

[3. Supporting Tables	24](#_Toc17807)

[4. Supporting References	37](#_Toc1260)

# 1. Experimental section

1.1. **Preparation of catalysts**
     1.1.1. **Synthesis of GaZrO_x_ via Sol-Gel Method**

GaZrO_x_ solid solutions were synthesized using a sol-gel process with gallium nitrate hydrate (Ga(NO_3_)_3_·xH_2_O), zirconium nitrate hydrate (Zr(NO_3_)_4_·5H_2_O), and glucose as precursors. The molar ratio of Ga to Zr was fixed at 0.5. Taking the catalyst calcined at a certain temperature. Specifically, 2.53 g of Ga(NO_3_)_3_·xH_2_O and 8.63 g of Zr(NO_3_)_4_·5H_2_O were dissolved in 150 mL of deionized water under continuous stirring in a 250 mL beaker at 80 °C in a water bath. After complete dissolution, 1.77 g of glucose was introduced into the homogeneous solution, followed by continuous stirring for 6 h to facilitate gel formation. The resulting gel was dried at 100 °C for 12 h, ground into powder, and then calcined for 4 h in a tubular furnace under air. Catalysts in this work were calcined at 600, 650, 700, 750, and 800 °C following the same procedure. The obtained samples are denoted as GZO-t, where t represents the calcination temperature.

1.1.2. **Synthesis of ZSM-5 and Ga-ZSM-5**

ZSM-5 and Ga-substituted ZSM-5 zeolites were synthesized via a hydrothermal method using tetraethyl orthosilicate (TEOS) as the silicon source, aluminum sulfate octadecahydrate (Al_2_(SO_4_)_3_·18H_2_O) as the aluminum source (or gallium nitrate hydrate as the gallium source), tetrapropylammonium hydroxide (TPAOH) as the structure-directing agent, and deionized water. The molar composition of the synthesis gel was ySiO_2_ : 0.5Al_2_O_3_ (or Ga_2_O_3_) : 0.27 TPAOH : 37 H_2_O. The gel was transferred into a 200 mL Teflon-lined stainless-steel autoclave and crystallized at 170 °C for 72 h, where y represents the Si/Al(Ga) ratio. The solid product was recovered by centrifugation, washed repeatedly with deionized water until neutral pH, dried and finally calcined at 540°C for 6 h in air to remove the organic template.

For the synthesis of ZSM-5 with Si/Al = 100, a typical procedure was as follows: Solution A was prepared by mixing 62.3 g of TEOS, 112.3 g of deionized water, and 65.7 g of TPAOH in a 500 mL beaker under stirring at 40 °C for 3 h. Solution B was obtained by dissolving 1.0 g of Al_2_(SO_4_)_3_·18H_2_O in 37.6 g of deionized water at 40 °C. Solution B was then added dropwise to Solution A under stirring, and the mixture was aged at 40 °C for 2 h. The resulting gel was hydrothermally treated as described above. ZSM-5 samples with different Si/Al ratios were synthesized by adjusting the amount of aluminum source accordingly and were denoted as Z5(y), where y represents the Si/Al or Si/Ga ratio. For Ga-ZSM-5, the aluminum source was replaced with Ga(NO_3_)_3_·xH_2_O while keeping other steps unchanged. The obtained materials are labeled as Ga-Z5(y).

1.1.3. **Preparation of Bifunctional Catalysts**

Bifunctional catalysts were fabricated via physical mixing. GaZrO_x_ and ZSM-5 (or Ga-ZSM-5) were combined in a 1:1 mass ratio and thoroughly homogenized in an agate mortar. The mixed powder was then pressed into pellets, crushed, and sieved to obtain particles with a mesh size of 20-40 for catalytic evaluations.

1.2. **Catalyst evaluation**

The catalytic performance of the GaZrO_x_/ZSM-5 composite catalyst was evaluated in a fixed-bed reactor. In each test, 1 g of catalyst was loaded in the middle of a stainless steel tube, secured in place with ceramic balls and quartz wool. A mixture of CO_2_/H_2_ with 1/4 molar ratio was introduced into the reactor. The pressure was increased to 3.0 MPa, and the total gas flow rate was set to 3600 mL/h, with a reaction temperature of 320 ℃. Unless otherwise specified, reactions were typically carried out at 320 ℃, 3.0 MPa, and a gas hourly space velocity (GHSV) of 3600 mL·g^-1^·h^-1^. The effluent gases, including inorganic species (CO_2_, CO, CH_4_), gasoline-range hydrocarbons, and gaseous hydrocarbons, were analyzed online using a gas chromatograph equipped with a flame ionization detector (FID) and a thermal conductivity detector (TCD). The CO_2_ conversion (Equation 1) and product selectivities (Equation 2) were calculated using the internal normalization method.

$${CO}_{2} conv.(\%)=\frac{n_{{CO}_{2},in}-n_{{CO}_{2},out}}{n_{{CO}_{2},in}}\times100\% (1)$$

$$S_{i} (\%)=\frac{m_{i,out}n_{i,out}}{n_{{CO}_{2},in}-n_{{CO}_{2},out}}\times100\% (2)$$

Here $n_{{CO}_{2},in}$ and $n_{{CO}_{2},out}$ are the molar concentrations of CO_2_ in the feedstock gas and effluent gas, respectively, $n_{i,out}$ is the concentration of product i, and $m_{i,out}$ is the number of carbon atoms of product i.

1.3. **Catalyst characterization**
     1.3.1. **X-ray Powder Diffraction (XRD).**

Phase structure characterization of the samples was performed using a Rigaku Smart Lab X-ray diffraction system. The instrument was equipped with a Cu Kα radiation source, operating at 40 kV and 100 mA. Data collection was conducted in continuous scan mode over a 2θ range of 5 ° to 80 °, with scan rates of 5 °/min or 8 °/min.

1.3.2. **X-ray Photoelectron Spectroscopy (XPS).**

Elemental analysis of the samples was carried out using a Thermo Scientific K-Alpha+ X-ray photoelectron spectrometer (XPS). The XPS spectra of O(1s), Ga(2p), and Zr(3d) were measured on an ESCALAB Xi+ DLD instrument with a monochromatic Al Kα X-ray source (hν = 1486.6 eV) under a base pressure of 5×10^-7^Pa. The binding energies were calibrated by referencing the adventitious carbon C(1s) peak to 284.8 eV.

1.3.3. **X-ray Absorption Spectroscopy (XAS).**

X-ray absorption spectroscopy (XAS) experiments for the Ga-ZSM-5 sample were performed at the BL11B beamline of the Shanghai Synchrotron Radiation Facility (SSRF). The XAS experiments for the GaZrO_x_ sample were conducted at the BL13SSW beamline of SSRF. The data were processed and fitted using the Demeter 0.9.26 software package with standard procedures. The Wavelet analysis was done using the HAMA Fortran software whereMorlet function with j = 10 and r = 1 was used.

1.3.4. **H_2_ Temperature-Programmed Reduction (H_2_-TPR).**

The H_2_ reduction capability of the samples was determined using a Quantachrome ChemBET Pulsar TPR/TPD chemisorption analyzer. Approximately 0.1 g of sample was placed in a U-shaped quartz tube. The sample was first purged with He at room temperature for 5 min to remove surface impurities. Subsequently, it was activated in a He flow by heating to 500 °C at a rate of 10 °C/min and holding for 1 h. After cooling to 40 °C, the gas was switched to H_2_, and the temperature was increased from 40 °C to 900 °C at 10 °C/min.

1.3.5. **CO_2_ Temperature-Programmed Desorption (CO_2_-TPD).**

The CO_2_ adsorption capacity of the samples was measured using a Quantachrome ChemBET Pulsar TPR/TPD chemisorption analyzer. Approximately 0.1 g of sample was placed in a U-shaped quartz tube. The sample was first purged with He at room temperature for 5 min. It was then activated in a He flow by heating to 500 °C at 10 °C/min and holding for 1 h. After cooling to 40 °C, the gas was switched to pure CO_2_ for adsorption treatment for 40 min. Subsequently, the sample was purged with He for 40 min to remove physisorbed CO_2_. Finally, the temperature was increased from 40 °C to 900 °C at a heating rate of 10 °C/min.

1.3.6. **H_2_-D_2_ Exchange.**

H_2_-D_2_ exchange experiments were performed using a Micromeritics Autochem II 2920 chemisorption analyzer equipped with a mass spectrometer. 50 mg of catalyst was loaded into a U-shaped sample tube. The sample was first purged with Ar at 30 mL/min, heated to 120 °C, and held for 30 min, then cooled to 50 °C. The gas was switched to He for purging for 30 min, followed by a mixture of 10% H_2_/Ar (40 mL/min) and D_2_ (4 mL/min) for 30 min. The temperature was then increased from 50 °C to 800 °C at 10 °C/min. The mass spectrometer monitored the changes in D_2_, H_2_, and HD (m/z = 3).

1.3.7. **Scanning Electron Microscopy (SEM).**

Sample morphology and grain size were observed with a Hitachi SU8220 cold field emission scanning electron microscope (SEM). A small amount of powder sample was ultrasonically dispersed in ethanol, dripped onto a silicon wafer, and dried naturally. The sample was then mounted on conductive tape on the sample stage. For secondary electron imaging, the accelerating voltage was 5-20 kV, the current was 10-20 μA, and the image resolution was 0.8 nm.

1.3.8. **Scanning Transmission Electron Microscopy and Energy Dispersive X-ray Spectroscopy (STEM-EDS).**

STEM was employed to observe the morphology, internal structure, and particle size distribution of the sample, while EDS was used to analyze the elemental distribution. The sample was tested using a JEOL JEM-F200 field-emission transmission electron microscope with an accelerating voltage of 200 kV. Prior to testing, a small amount of the powdered sample was ultrasonically dispersed in anhydrous ethanol, and a drop of the suspension was transferred onto a copper grid using a capillary tube. The sample was allowed to dry before analysis.

1.3.9. **Physical Adsorption-Desorption.**

Specific surface area and pore structure characteristics of the samples were determined using an Autosorb-SI physisorption analyzer. Prior to analysis, approximately 50 mg of sample was degassed at 300 °C (10^-3^ Pa) for 6-8 h. After cooling to room temperature, the pores were filled with N_2_ or Ar. The adsorption isotherms were measured at liquid nitrogen temperature (-196 °C) or liquid argon temperature (-186 °C). Due to its high surface area, the Ga-ZSM-5 sample was tested using N_2_ as the adsorbate, while Ar was used for GaZrO_x_.

The specific surface area of the samples was calculated using the Brunauer-Emmett-Teller (BET) multipoint equation. The micropore surface area and micropore volume were determined by the t-plot method. The total pore volume was obtained from the adsorption data at a relative pressure (P/P_0_) of 0.95. Pore size distribution and other characteristics derived from the N_2_ and Ar physical adsorption-desorption isotherms were analyzed using the Barrett-Joyner-Halenda (BJH) method and the Non-Local Density Functional Theory (NLDFT) method, respectively.

1.3.10. **NH_3_ Temperature-Programmed Desorption (NH_3_-TPD).**

NH_3_-TPD experiments were conducted on a Quantachrome ChemBET Pulsar TPR/TPD chemisorption analyzer to investigate the strength, amount, and distribution of acid sites. Approximately 0.1 g of sample was placed in a U-shaped quartz tube. The sample was pretreated in a He flow at 500 °C for 1 h for purification, then cooled to 393 K. After temperature stabilization, the gas was switched to an 8 vol% NH_3_/He mixture for ammonia adsorption at 393 K for 1 h. Following chemisorption, the sample was purged with He for 1 h to remove physisorbed NH_3_. The temperature was then increased to 873 K at a rate of 10 K/min under He flow for the desorption process. The desorbed NH_3_ was monitored in real-time using a thermal conductivity detector (TCD). The total number of acid sites was quantified based on a standard NH_3_-TPD calibration curve.

1.3.11. **Pyridine Adsorption Infrared Spectroscopy (Py-IR).**

The concentrations of Brønsted and Lewis acid sites were determined by pyridine adsorption infrared spectroscopy (Py-IR) using a Bruker EQUINOX-55 Fourier transform infrared spectrometer. A small amount of powder sample was pressed into a self-supporting wafer (~20 mg, 12.5 mg·cm^-2^) and placed in a pyridine adsorption cell. The sample was heated from room temperature to 450 °C at 10 °C/min and held for 1 h under vacuum to remove surface impurities. After cooling to room temperature, a background spectrum was collected. The sample was then exposed to pyridine vapor for 15 min under vacuum. After pyridine adsorption, the temperature was raised to 150 °C and held for 30 min, then cooled to room temperature for Py-IR desorption spectra collection. This was repeated after heating to 350 °C for 30 min.

1.3.12. **In Situ Diffuse Reflectance Infrared Fourier Transform Spectroscopy (*In Situ* DRIFTS).**

Adsorbed species and reaction intermediates on the samples were investigated using a Thermo Nicolet iS50 FTIR spectrometer equipped with an in situ DRIFTS cell and a liquid nitrogen-cooled mercury cadmium telluride (MCT) detector. The sample was loaded into the in situ cell. The pretreatment was conducted at a fixed pressure of 3 MPa. The catalyst was heated to 400 °C at 10 °C/min under N_2_ flow and held for 1 h. The temperature was then lowered to room temperature, and the gas was switched to H_2_ for purging for 15 min. A background spectrum was collected. Subsequently, the temperature was increased to 450 °C while continuously collecting spectra to monitor surface species. After the temperature was lowered to 320 °C, a CO_2_/H_2_ mixture (CO_2_/H_2_ = 1/4) was introduced and maintained for 1 h, with continuous spectra collection to identify reaction intermediates.

**1.3.13 Inductively Coupled Plasma Optical Emission Spectrometry (ICP-OES).**

The contents of Si and Ga elements in the Ga-ZSM-5 sample were determined using a PerkinElmer Avio 500 inductively coupled plasma optical emission spectrometer. Prior to analysis, the solid sample was digested into a clear solution using hydrochloric acid or hydrofluoric acid. The specific content of each element was then measured and calculated.

**1.3.14 Thermogravimetric Analysis (TGA).**

Thermogravimetric analysis of the samples was performed using an SDT Q-600 thermogravimetric analyzer (TA Instruments, USA). About 10 mg of sample was heated from room temperature to 900 °C at a rate of 10 °C·min^-1^ under an air flow of 100 mL·min^-1^. The weight change and weight loss rate of the sample were recorded.

# 2. Supporting Figures

****Fig. S1**.** TG curves of (a) GZO-700/Z5(50) after 240 h on stream and (b) GZO-700/Ga-Z5(50) after 400 h on stream.

****Fig. S2.**** The shift of XRD diffraction peaks of GaZrO_x_-700 relative to t-ZrO_2_

****Fig. S3.**** (a) Argon physisorption/desorption isotherms and (b) pore size distribution profiles of GaZrO_x_ catalysts calcined at different temperatures.

****Fig. S4.**** TG/DTG profiles of the as-synthesized GaZrO_x_ precursor before calcination.

****Fig. S5.**** CO_2_ temperature-programmed desorption (CO_2_-TPD) profiles of GaZrO_x_ catalysts calcined at different temperatures.

****Fig. S6.**** H_2_ temperature-programmed reduction (H_2_-TPR) profiles of GaZrO_x_ catalysts calcined at different temperatures.

****Fig. S7.**** (a) Zr K-edge XANES spectra and (b) Fourier transform of the Zr K-edge EXAFS spectra.

****Fig. S8.**** (a-e) Fourier-transformed (FT) magnitude of k^3^-weighted Zr K-edge EXAFS spectra in R-space and the corresponding EXAFS fitting results for GaZrO_x_ catalysts calcined at different temperatures.

****Fig. S9.**** (a-e) Zr K-edge EXAFS oscillation functions k^3^χ(k) in k-space and their corresponding EXAFS fitting results for GaZrO_x_ catalysts calcined at different temperatures.

**Fig. S10.** (a-b) Wavelet transforms of Ga K-edge EXAFS spectra for the GZO-650 and GZO-750 samples.

**Fig. S11.** (a-e) Fourier-transformed (FT) magnitude of k^3^-weighted Ga K-edge EXAFS spectra in R-space and the corresponding EXAFS fitting results for GaZrOx catalysts calcined at different temperatures.

**Fig. S12.** (a-e) Ga K-edge EXAFS oscillation functions k^3^χ(k) in k-space and their corresponding EXAFS fitting results for GaZrOx catalysts calcined at different temperatures.

**Fig. S13.** (a-c) In situ DRIFTS spectra of GZO calcined at 600, 700, and 800 °C; reaction conditions: 3 MPa, 320 °C, CO_2_ + H_2_ atmosphere.

****Fig. S14.**** Scanning electron microscopy (SEM) images of (a) ZSM-5(50), (b) ZSM-5(100), (c) ZSM-5(200), and (d) ZSM-5(300).

****Fig. S15.**** Scanning electron microscopy (SEM) images of Ga-ZSM-5 samples: (a) Ga-Z5(20), (b) Ga-Z5(50), and (c) Ga-Z5(100).

****Fig. S16.**** XRD patterns of ZSM-5 with different Si/Al ratios.

****Fig. S17.**** (a) N_2_ physisorption isotherms and (b) pore size distribution profiles of ZSM-5 zeolites with different Si/Al ratios.

****Fig. S18.**** (a) N_2_ physisorption/desorption isotherms and (b) pore size distribution profiles of Ga-ZSM-5 samples.

****Fig. S19**.** R-space (a-c) experiments and fitting profiles of the Ga-Z5(20), Ga-Z5(50) and Ga-Z5(100).

****Fig. S20**.** k-space (a-c) experiments and fitting profiles of the Ga-Z5(20), Ga-Z5(50) and Ga-Z5(100).

****Fig. S21**.** Ammonia temperature-programmed desorption (NH_3_-TPD) profiles of ZSM-5 with different Si/Al ratios

# 3. Supporting Tables

**Table S1.** CO_2_ conversion, CO selectivity, and hydrocarbon distribution over GaZrO_x_/Z5(100) tandem catalysts.

| Catalysts | CO_2_  conv.  (%) | CO  sel.  (%) | CH_4_  (%) | C_2_  (%) | C_3_  (%) | C_4_  (%) | C_5+_  (%) |
| --- | --- | --- | --- | --- | --- | --- | --- |
| GZO-600/Z5(100) | 4.4 | 20.9 | 0.6 | 8.2 | 10.9 | 10.2 | 70.0 |
| GZO-650/Z5(100) | 11.4 | 16.9 | 1.7 | 10.5 | 10.4 | 7.6 | 69.8 |
| GZO-700/Z5(100) | 13.5 | 12.3 | 1.1 | 5.0 | 7.6 | 13.3 | 73.0 |
| GZO-750/Z5(100) | 6.3 | 14.6 | 0.6 | 7.4 | 10.2 | 11.1 | 70.8 |
| GZO-800/Z5(100) | 1.7 | 15.7 | 1.3 | 5.5 | 10.4 | 16.0 | 66.8 |

Reaction conditions: 1 g catalysts, molar ratio of H_2_/CO_2_ = 4/1, P = 3 MPa, T = 320 °C, GHSV = 3600 mL·g^-1^·h^-1^.

**Table S2.** CO_2_ conversion, CO selectivity, and hydrocarbon distribution over GZO-700/ZSM-5 tandem catalysts.

| Catalysts | CO_2_  conv.  (%) | CO  sel.  (%) | CH_4_  (%) | C_2_  (%) | C_3_  (%) | C_4_  (%) | C_5+_  (%) |
| --- | --- | --- | --- | --- | --- | --- | --- |
| GZO-700/Z5(50) | 13.1 | 12.4 | 1.3 | 3.7 | 9.7 | 18.8 | 66.5 |
| GZO-700/Z5(100) | 13.5 | 12.3 | 1.1 | 5 | 7.6 | 13.3 | 73.0 |
| GZO-700/Z5(200) | 14.7 | 11.9 | 0.8 | 5.3 | 5.8 | 11.6 | 76.5 |
| GZO-700/Z5(300) | 14.6 | 12.9 | 0.9 | 7.6 | 6.1 | 9.2 | 76.2 |

Reaction conditions: 1 g catalysts, molar ratio of H_2_/CO_2_ = 4/1, P = 3 MPa, T = 320 °C, GHSV = 3600 mL·g^-1^·h^-1^.

Table S3 Detailed products distribution of C_5+_ products on GZO-700/Z5(200) for CO_2_ hydrogenation

| Product | Proportion (%) |
| --- | --- |
| C_5_ aliphatics | 15.5 |
| C_6_ aliphatics | 19.7 |
| C_7_ aliphatics | 8.1 |
| Toluene | 7 |
| Ethylbenzene | 3.7 |
| p-Xylene | 4.4 |
| m-Xylene | 4.5 |
| o-Xylene | 5.2 |
| Methyltriethylbenzene | 0.9 |
| 1,2,4-Trimethylbenzene | 1.9 |
| Mesitylene(1,3,5-Trimethylbenzene) | 8.9 |
| 1,2,3,5-Tetramethylbenzene | 0.7 |
| 1,2,4,5-Tetramethylbenzene | 18.8 |
| 1,2,3,4-Tetramethylbenzene | 0.7 |

**Table S4.** CO_2_ conversion, CO selectivity, and hydrocarbon distribution over GZO-700/Ga-ZSM-5 tandem catalysts.

| Catalysts | CO_2_  conv.  (%) | CO  sel.  (%) | CH_4_  (%) | C_2_  (%) | C_3_  (%) | C_4_  (%) | C_5+_  (%) |
| --- | --- | --- | --- | --- | --- | --- | --- |
| GZO-700/Ga-Z5(20) | 9.4 | 13.4 | 2.2 | 2.4 | 4.8 | 22.1 | 68.6 |
| GZO-700/Ga-Z5(50) | 17.2 | 11.7 | 0.7 | 3.6 | 4.2 | 8.4 | 83.2 |
| GZO-700/Ga-Z5(100) | 16.4 | 14.3 | 0.9 | 8.7 | 7.8 | 7.7 | 74.9 |

Reaction conditions: 1 g catalysts, molar ratio of H_2_/CO_2_ = 4/1, P = 3 MPa, T = 320 °C, GHSV = 3600 mL·g^-1^·h^-1^.

**Table S5.** Exploration on the packing mode of GZO-700/Ga-Z5(50) tandem catalyst.

| Catalysts loading method | CO_2_  conv.  (%) | CO  sel.  (%) | CH_4_  (%) | C_2_  (%) | C_3_  (%) | C_4_  (%) | C_5+_  (%) |
| --- | --- | --- | --- | --- | --- | --- | --- |
| Dual bed | 10.7 | 15.1 | 1.1 | 2.6 | 5.4 | 18.4 | 72.0 |
| Granule mixing | 13.1 | 9.9 | 0.9 | 4.2 | 6.6 | 17.6 | 70.5 |
| Powder mixing | 17.2 | 11.7 | 0.7 | 3.6 | 4.2 | 8.4 | 83.2 |

Reaction conditions: 1 g catalysts, molar ratio of H_2_/CO_2_ = 4/1, P = 3 MPa, T = 320 °C, GHSV = 3600 mL·g^-1^·h^-1^.

**Table S6.** Comparison of reported work on CO_2_ to C_5+_ based on CO_2_-MeOH route.

| Catalysts | T (^o^C) | P (MPa) | GHSV (mL·g^-1^·h^-1^) | CO_2_  Conv. (%) | | CO  Sel.  (%) | CH_4_  Sel. (%) | | C_5+_  Sel. (%) | C_5+_  Yield (%) | Ref. |
| --- | --- | --- | --- | --- | --- | --- | --- | --- | --- | --- | --- |
| ZnAlO_x_&H-ZSM-5 | 320 | 3.0 | 2000 | 9.1 | 57.4 | | 0.4 | 79.7 | | 3.1 | [1] |
| 1Co-In_2_O_3_-ZrO_2_/HZSM-5 | 300 | 3.0 | 3600 | 6.05 | 37.0 | | ~2 | 66.2 | | 2.5 | [2] |
| In_2_O_3_/HZSM-5 | 340 | 4.0 | 9000 | 13.1 | 44.5 | | 1 | 78.6 | | 5.7 | [3] |
| ZnO/ZrO_2_-Z5-300 | 340 | 3.0 | 2700 | 9.1 | 42.5 | | 0.6 | 70.0 | | 3.7 | [4] |
| ZnZr_8_O(350)-Zn/Z5 | 320 | 3.0 | 4800 | 15.2 | 41.3 | | ~5.0 | 75.7 | | 6.8 | [5] |
| ZnCrO_x_-ZnZSM-5 | 320 | 5.0 | 2000 | 19.9 | 70.2 | | 30.3 | 69.7 | | 4.1 | [6] |
| Cr_2_O_3_/Zn-ZSM-5@SiO_2_ | 350 | 3.0 | 1200 | 22.1 | 35.1 | | 4.5 | 63.4 | | 9.1 | [7] |
| In_2_O_3_/HZC-2.5 | 340 | 3.0 | 6000 | 22.7 | 52.5 | | - | 76.2 | | 8.2 | [8] |
| ZZ/Z5-0.73 | 315 | 3.0 | 1020 | 17.5 | 23.8 | | 1.2 | 75.1 | | 10.0 | [9] |
| ZnZrO_x_/ZSM-5-300 | 320 | 4.16 | 7200 | ~11.0 | ~38.0 | | - | 62.7 | | 4.3 | [10] |
| ZnCrO_x_/H-ZSM-5(200) | 330 | 3.0 | 3000 | 17.5 | 38.1 | | ~0.5 | 69.9 | | 7.6 | [11] |
| ZrO_2_-Cr(8:1)/HZSM-5@SiO_2_ | 390 | 3.0 | 1200 | 13.9 | 45.1 | | 1.1 | 80.1 | | 6.1 | [12] |
| GaZrO_x_-700/Ga-Z5(50) | 320 | 3.0 | 3600 | 17.2 | 11.7 | | 0.7 | 83.2 | | 12.6 | This work |

****Table S7.** Pore structure parameters of GaZrO_x_ catalysts calcined at different temperatures.**

| Samples | S_BET_ ^[a]^ (m^2^·g^-1^) | V_Total_ ^[b]^ (cm^3^·g^-1^) | V_Meso_ ^[c]^ (cm^3^·g^-1^) |
| --- | --- | --- | --- |
| GZO-600 | 22 | 0.022 | 0.022 |
| GZO-650 | 46 | 0.054 | 0.054 |
| GZO-700 | 22 | 0.038 | 0.038 |
| GZO-750 | 15 | 0.039 | 0.039 |
| GZO-800 | 13 | 0.0082 | 0.0082 |

^[a]^ BET method, ^[b]^ V-t method, ^[c]^ V_Total_ = V_Meso_.

**Table S8.** O 1s XPS peak deconvolution results.

| Samples | O_lattice_ (%) | O_defect_ (%) | OH (%) | H_2_O (%) |
| --- | --- | --- | --- | --- |
| GZO-600 | 55.1 | 18.0 | 18.1 | 8.8 |
| GZO-650 | 62.6 | 19.5 | 10.4 | 7.5 |
| GZO-700 | 56.4 | 31.4 | 7.5 | 4.7 |
| GZO-750 | 63.0 | 22.9 | 10.3 | 3.8 |
| GZO-800 | 65.1 | 15.1 | 12.2 | 7.6 |

**Table S9.** Curve fit Parameters ^[a]^ for Zr K-edge for GaZrO_x_ samples.

| Samplest | path | N | R/Å | ^[b]^ σ^2^/Å^2^ | R-factor |
| --- | --- | --- | --- | --- | --- |
| GZO-600 | Zr-O_1_ | 3.8 ± 0.9 | 2.1 ± 0.02 | 0.002 ± 0.003 ^[c]^ | 0.01 |
|  | Zr-O_2_ | 2.7 ± 0.4 | 2.3 ± 0.03 | 0.002 ± 0.003 ^[c]^ |  |
| GZO-650 | Zr-O_1_ | 3.9 ± 1.0 | 2.1 ± 0.02 | 0.002 ± 0.003 ^[c]^ | 0.03 |
|  | Zr-O_2_ | 2.5 ± 0.5 | 2.3 ± 0.03 | 0.002 ± 0.003 ^[c]^ |  |
| GZO-700 | Zr-O_1_ | 4.3 ± 1.2 | 2.1 ± 0.02 | 0.004 ± 0.003 ^[c]^ | 0.03 |
|  | Zr-O_2_ | 2.4 ± 0.6 | 2.3 ± 0.04 | 0.004 ± 0.003 ^[c]^ |  |
| GZO-750 | Zr-O_1_ | 4.3 ± 1.1 | 2.1 ± 0.02 | 0.003 ± 0.003 ^[c]^ | 0.02 |
|  | Zr-O_2_ | 2.3 ± 0.6 | 2.3 ± 0.04 | 0.003 ± 0.003 ^[c]^ |  |
| GZO-800 | Zr-O_1_ | 4.3 ± 1.1 | 2.1 ± 0.02 | 0.003 ± 0.003 ^[c]^ | 0.02 |
|  | Zr-O_2_ | 2.2 ± 0.6 | 2.3 ± 0.04 | 0.003 ± 0.003 ^[c]^ |  |

^[a]^ s_0_^2^ was fixed as 0.81. Data ranges of GZO-600 sample: 3 Å^-1^ ≤ k ≤ 12 Å^-1^, 1 Å ≤ R ≤ 2.5 Å, other samples: 3 Å^-1^ ≤ k ≤ 12 Å^-1^, 1 Å ≤ R ≤ 4 Å. The number of variable parameters for sample GZO-600 is 6, out of a total of 8.4 independent data points; while that for the other samples is 9, out of a total of 17.0 independent data points. ^[b]^ σ^2^ is the Debye-Waller factor. ^[c]^ The Debye-Waller factors were constrained as σ^2^(Zr-O_1_) = σ^2^(Zr-O_2_) to reduce the correlation (or the number of variables). R-factor is residual factor.

**Table S10.** Curve fit Parameters ^[a]^ for Ga K-edge for GaZrO_x_ samples.

| Samplest | path | N | R/Å | σ^2^/Å^2^ | R-factor |
| --- | --- | --- | --- | --- | --- |
| GZO-600 | Ga-O | 4.5 ± 0.7 | 1.87 ± 0.02 | 0.007 ± 0.002 | 0.006 |
| GZO-650 | Ga-O | 4.9 ± 0.7 | 1.89 ± 0.01 | 0.008 ± 0.002 | 0.006 |
| GZO-700 | Ga-O | 5.0 ± 0.8 | 1.89 ± 0.02 | 0.008 ± 0.003 | 0.007 |
| GZO-750 | Ga-O | 5.0 ± 0.9 | 1.89 ± 0.02 | 0.008 ± 0.003 | 0.007 |
| GZO-800 | Ga-O | 4.9 ± 0.9 | 1.88 ± 0.02 | 0.008 ± 0.003 | 0.007 |

^[a]^ s_0_^2^ was fixed as 0.89. Data ranges: 3 Å^-1^ ≤ k ≤ 9 Å^-1^, 1 Å ≤ R ≤ 2.5 Å.The number of variable parameters is 4, out of a total of 6.5 independent data points. σ^2^ is the Debye-Waller factor. R-factor is residual factor.

**Table S11.** ICP results of Ga‑ZSM-5.

| Sample | Ga-Z5(20) | Ga-Z5(50) | Ga-Z5(100) |
| --- | --- | --- | --- |
| ICP | Si/Ga = 31 | Si/Ga = 74 | Si/Ga = 160 |

****Table S12.**** Pore structural parameters of ZSM-5 catalysts with different Si/Al ratios.

| Samples | S_BET_ ^[a]^  (m^2^·g^-1^) | S_Micro_ ^[b]^  (m^2^·g^-1^) | V_Total_ ^[c]^  (cm^3^·g^-1^) | V_Micro_ ^[d]^  (cm^3^·g^-1^) | V_Meso_ ^[e]^  (cm^3^·g^-1^) |
| --- | --- | --- | --- | --- | --- |
| Z5(50) | 409 | 370 | 0.22 | 0.16 | 0.06 |
| Z5(100) | 438 | 416 | 0.21 | 0.18 | 0.03 |
| Z5(200) | 435 | 391 | 0.24 | 0.16 | 0.08 |
| Z5(300) | 448 | 414 | 0.24 | 0.18 | 0.06 |

^[a]^ BET method, ^[b]^ V-t method, ^[c]^ P/P_0_ = 0.95, ^[d]^ t-plot method, ^[e]^ V_Total_-V_Micro_.

****Table S13.**** Pore structural parameters of the Ga-ZSM-5 catalysts.

| Samples | S_BET_ ^[a]^  (m^2^·g^-1^) | S_Micro_ ^[b]^  (m^2^·g^-1^) | V_Total_ ^[c]^  (cm^3^·g^-1^) | V_Micro_ ^[d]^  (cm^3^·g^-1^) | V_Meso_ ^[e]^  (cm^3^·g^-1^) |
| --- | --- | --- | --- | --- | --- |
| Ga-Z5(20) | 408 | 339 | 0.32 | 0.14 | 0.18 |
| Ga-Z5(50) | 441 | 398 | 0.23 | 0.17 | 0.06 |
| Ga-Z5(100) | 444 | 415 | 0.23 | 0.18 | 0.05 |

^[a]^ BET method, ^[b]^ V-t method, ^[c]^ P/P_0_ = 0.95, ^[d]^ t-plot method, ^[e]^ V_Total_-V_Micro_.

**Table S14.** Curvefit Parameters ^[a]^ for Ga K-edge for Ga-ZSM-5 samples.

| Samples | path | N | R/Å | σ^2^/Å^2^ | R-factor |
| --- | --- | --- | --- | --- | --- |
| Ga-Z5(20) | Ga-O | 3.9 ± 0.2 | 1.81 ± 0.01 | 0.002 ± 0.001 | 0.003 |
| Ga-Z5(50) | Ga-O | 4.1 ± 0.3 | 1.80 ± 0.01 | 0.002 ± 0.001 | 0.004 |
| Ga-Z5(100) | Ga-O | 4.2 ± 0.3 | 1.81 ± 0.01 | 0.002 ± 0.001 | 0.006 |

^[a]^ s_0_^2^ was fixed as 0.78. Data ranges: 3 Å^-1^ ≤ k ≤ 12.5 Å^-1^, 1 Å ≤ R ≤ 2 Å. The number of variable parameters is 4, out of a total of 5.9 independent data points. σ^2^ is the Debye-Waller factor. R-factor is residual factor.

# 4. Supporting References

[1] Y. Ni, Z. Chen, Y. Fu, Y. Liu, W. Zhu, Z. Liu, “Selective conversion of CO_2_ and H_2_ into aromatics” *Nat. Commun.* **9**, (2018): 3457.

[2] W. Li, J. Zhang, X. Jiang, M. Mu, A. Zhang, C. Song, X. Guo, “Co-Promoted In_2_O_3_/ZrO_2_ Integrated with Ultrathin Nanosheet HZSM-5 as Efficient Catalysts for CO_2_ Hydrogenation to Gasoline” *Ind. Eng. Chem. Res.* **61**, (2022): 6322-6332.

[3] P. Gao, S. Li, X. Bu, S. Dang, Z. Liu, H. Wang, L. Zhong, M. Qiu, C. Yang, J. Cai, W. Wei, Y. Sun, “Direct conversion of CO_2_ into liquid fuels with high selectivity over a bifunctional catalyst” *Nat. Chem.* **9**, (2017): 1019-1024.

[4] X. Zhang, A. Zhang, X. Jiang, J. Zhu, J. Liu, J. Li, G. Zhang, C. Song, X. Guo, “Utilization of CO_2_ for aromatics production over ZnO/ZrO_2_-ZSM-5 tandem catalyst” *J. CO_2_ Util.* **29**, (2019): 140-145.

[5] H. Tian, J. Jiao, F. Zha, X. Guo, X. Tang, Y. Chang, H. Chen, “Hydrogenation of CO_2_ into aromatics over ZnZrO_x_–Zn/HZSM-5 composite catalysts derived from ZIF-8” *Catal. Sci. Technol.* **12**, (2022): 799-811.

[6] J. Zhang, M. Zhang, S. Chen, X. Wang, Z. Zhou, Y. Wu, T. Zhang, G. Yang, Y. Han, Y. Tan, “Hydrogenation of CO_2_ into aromatics over a ZnCrO_x_-zeolite composite catalyst” *Chem. Commun.* **55**, (2019): 973-976.

[7] Y. Wang, W. Gao, S. Kazumi, H. Li, G. Yang, N. Tsubaki, “Direct and oriented conversion of CO_2_ into value-added aromatics” *Chem. Eur. J.* **25**, (2019): 5149-5153.

[8] H. Tian, J. Luo, Y. Li, M. Cao, F. Zha, Y. Chang, H. Chen, “Effects of HZSM-5 zeolite surface hydrophobicity on CO_2_ hydrogenation to gasoline” *Sep. Purif. Technol*. **386**, (2026): 136607.

[9] T. Wang, C. Yang, P. Gao, S. Zhou, S. Li, H. Wang, Y. Sun, “ZnZrO_x_ integrated with chain-like nanocrystal HZSM-5 as efficient catalysts for aromatics synthesis from CO_2_ hydrogenation” *Appl. Catal. B: Environ.* **286**, (2021): 119929.

[10] I. Nezam, W. Zhou, D. Shah, M. Bukhovko, M. Ball, G. Gusmão, A. Medford, C. Jones, “Role of catalyst domain size in the hydrogenation of CO_2_ to aromatics over ZnZrO_x_/ZSM-5 catalysts” *J. Phys. Chem. C.* **127**, (2023): 6356-6370.

[11] S. Guo, S. Fan, H. Wang, S. Wang, Z. Qin, M. Dong, W. Fan, J. Wang, “Selective conversion of CO_2_ to trimethylbenzene and ethene by hydrogenation over a bifunctional ZnCrO_x_/H-ZSM-5 composite catalyst” *ACS Catal.* **14**, (2023): 271-282.

[12] L. Zhang, W. Gao, F. Wang, C. Wang, J. Liang, X. Guo, Y. He, G. Yang, N. Tsubaki, “Highly selective synthesis of light aromatics from CO_2_ by chromium-doped ZrO_2_ aerogels in tandem with HZSM-5@SiO_2_ catalyst” *Appl. Catal. B: Environ.* **328**, (2023): 122535.
